# The Antiviral Small-Interfering RNA Pathway Induces Zika Virus Resistance in Transgenic *Aedes aegypti*

**DOI:** 10.3390/v12111231

**Published:** 2020-10-30

**Authors:** Adeline E. Williams, Irma Sanchez-Vargas, William R. Reid, Jingyi Lin, Alexander W.E. Franz, Ken E. Olson

**Affiliations:** 1 Department of Microbiology, Immunology, and Pathology, Colorado State University, Fort Collins, CO 80523, USA; adeline.williams@colostate.edu (A.E.W.); irma.sanchez-vargas@colostate.edu (I.S.-V.); 2 Department of Veterinary Pathobiology, University of Missouri, Columbia, MO 65211, USA; reidwi@missouri.edu (W.R.R.); linjin@missouri.edu (J.L.); franza@missouri.edu (A.W.E.F.)

**Keywords:** *Aedes aegypti*, Zika virus, transgenesis, RNAi, siRNAs, antiviral immunity, arbovirus, CRISPR/Cas9

## Abstract

The resurgence of arbovirus outbreaks across the globe, including the recent Zika virus (ZIKV) epidemic in 2015–2016, emphasizes the need for innovative vector control methods. In this study, we investigated ZIKV susceptibility to transgenic *Aedes aegypti* engineered to target the virus by means of the antiviral small-interfering RNA (siRNA) pathway. The robustness of antiviral effector expression in transgenic mosquitoes is strongly influenced by the genomic insertion locus and transgene copy number; we therefore used CRISPR/Cas9 to re-target a previously characterized locus (Chr2:321382225) and engineered mosquitoes expressing an inverted repeat (IR) dsRNA against the NS3/4A region of the ZIKV genome. Small RNA analysis revealed that the IR effector triggered the mosquito’s siRNA antiviral pathway in bloodfed females. Nearly complete (90%) inhibition of ZIKV replication was found in vivo in both midguts and carcasses at 7 or 14 days post-infection (dpi). Furthermore, significantly fewer transgenic mosquitoes contained ZIKV in their salivary glands (*p* = 0.001), which led to a reduction in the number of ZIKV-containing saliva samples as measured by transmission assay. Our work shows that *Ae. aegypti* innate immunity can be co-opted to engineer mosquitoes resistant to ZIKV.

## 1. Introduction

Arthropod-borne (arbo) viruses such as Zika (ZIKV; *Flaviviridae*; *Flavivirus*), dengue 1–4 (DENV1-4; *Flaviviridae*; *Flavivirus*), and chikungunya (CHIKV; *Togaviridae*; *Alphavirus*) are major public health burdens that continue to threaten hundreds of millions of people worldwide [1,2]. The main vector of these viruses is the urban dwelling, anthropophilic *Aedes aegypti* mosquito. Few to no vaccines or antiviral therapeutics are available for most arboviruses, including ZIKV, and limiting human exposure to infected mosquitoes remains the primary method for preventing disease transmission. Conventional vector control involves sustained insecticide usage, which is expensive, laborious, and selects for insecticide resistance [3]. Novel strategies to manage and prevent mosquito-borne diseases are therefore desperately needed [4].

The generation of genetically modified mosquitoes to reduce disease burdens has been proposed as a vector control strategy that could complement currently implemented methods [5,6,7]. In 2016, for example, the WHO Zika virus research agenda (WHO reference number WHO/ZIKV/PHR/16.1) announced their support for new vector control tools for ZIKV, including transgenic mosquitoes. In addition, transgenic *Ae. aegypti* mosquitoes from Oxitec (Oxford, UK) have been tested for population suppression in Brazil and the Grand Cayman Islands and have recently been approved for use in the Florida Keys [8,9,10,11,12,13]. Taken together, this represents a burgeoning paradigm shift towards genetic pest management of the *Ae. aegypti* disease vector.

In addition to population suppression genetic technologies, a population replacement strategy involves the development of transgenic mosquitoes refractory to arboviruses that harbor two essential transgene components—the antiviral effector, which is intended to eliminate viral infection in the mosquito before it can be transmitted to a new host, and the gene drive element, which is intended to overcome any fitness costs associated with the antiviral effector element [14,15,16,17]. This then would ensure that the effector cargo can reach fixation in a targeted wild-type population [18].

We previously found that synthetic resistance to arboviruses in *Ae. aegypti* could be achieved by co-opting the innate immune responses of the mosquito by utilizing its RNA interference (RNAi) pathway [19]. Transgenic *Ae. aegypti* expressing an inverted repeat (IR) RNA targeting the pre-membrane region of the DENV2 genome triggered the small-interfering RNA (siRNA) pathway, resulting in ~100% DENV2 resistance [19,20,21]. In order to generate transgenic DENV2-refractory *Ae. aegypti*, the *mariner Mos1* transposon was used. However, the use of Class II DNA transposons such as *mariner Mos1*, *piggyBac*, or *Hermes* [22,23,24] for transposon-mediated genome insertion is quasi-random and prone to insertional position effects [20,25].

Assessment of several DENV2-targeting transgenic *Ae. aegypti* lines that differed from each other only by their genomic transgene insertion sites displayed a range in both effectiveness of DENV2 suppression and transgene stability [20,26]. Most notably, while multiple transgenic lines demonstrated high levels of DENV2 suppression, one of these lines lost its resistance phenotype after only 17 generations in laboratory colony. Meanwhile, another transgenic line has remained refractory to DENV2 for >54 generations [20]. Given the high impact of transgene genomic insertion site on both expression levels and long-term stability of antiviral effectors, this aspect should always be taken into consideration when engineering transgenic resistance to pathogens in *Ae. aegypti*.

For our study, we chose a genomic locus that was previously identified in Dong et al. (2017) to have robust and stable transgene expression in the midgut [27,28]. In that study, Dong et al. (2017) used *mariner Mos1* to overexpress a tissue inhibitor of metalloproteinases (TIMP) transgene under control of the *carboxypeptidase A* promoter in the female midgut following bloodmeal ingestion. Because transgene insertion by way of the *mariner Mos1* transposon is quasi-random, this method allows for the discovery of novel genomic loci that are permissive for strong and stable transgene expression. Indeed, Dong et al. (2017) identified a total of seven unique insertion sites that resulted in three viable transgenic lines, among which one line displayed strong and robust transgene expression in the midgut. Functional testing of the transgenic mosquito line demonstrated a significant increase in the dissemination of chikungunya virus, which proved that the inserted transgene had the expected biological activity in the mosquito midgut. Further, the authors mapped the inserted transgene to a single intergenic locus on chromosome 2 and performed genetic outcrosses to confirm single locus insertion. Recently, work in our laboratory has shown that the transgenic line from Dong et al. (2017) retains stable transgene expression for more than 10 generations (unpublished). Given that the chromosome 2 locus resulted in strong and stable transgene expression in the midgut, we used the CRISPR/Cas9 system to introduce our anti-ZIKV effector proximal to the identified *mariner Mos1* insertion site.

For construction of the IR effector, we selected a cDNA sequence derived from the NS3/4A region of the ZIKV genome, which has recently been identified to be a highly effective target at suppressing the virus when transiently tested in *Ae. aegypti* [29]. In addition, while the approach to insert a transgene into a specific genome locus allows for robust transgene expression, it also allows for the direct side-by-side comparison and optimization of other antiviral effectors since any insertional position effects are cancelled out between them.

## 2. Materials and Methods

### 2.1. Mosquito Rearing and Maintenance

All *Ae. aegypti* mosquito colonies were maintained at 28 °C with 75–80% relative humidity and a 12 h light/12 h dark cycle. Routine maintenance regimens are described in [20]. Briefly, mated females were fed artificial bloodmeals consisting of defibrinated sheep blood (Colorado Serum Co., Denver, CO, USA) and 10 mM ATP approximately 4 days post emergence. Females were encaged with oviposition cups (consisting of paper towel strips and small water-filled plastic cups) for 5 days, and the eggs were then retrieved and dried. Stored eggs were viable for up to 3 months. Eggs were hatched in sterile water, and larvae were fed with ground TetraMin (Melle, Germany) fish food.

### 2.2. Identification of Active sgRNA Target Sites

CHOPCHOP [30,31] was used to design four sgRNAs as close to the original *mariner Mos1* insertion site [28] as possible while avoiding any predicted off-target sequences. The genomic DNA from a pool of 10 female and 10 male *Ae. aegypti* (HWE strain [32]) was sequenced across the locus containing the sgRNA target sites to confirm their presence and integrity. Each of the sgRNAs was then tested for DNA cleavage activity in the mosquito embryo by injection of three sets of ~100 *Ae. aegypti* embryos for each sgRNA. The injection mixes contained 300 ng/µL Cas9-NLS protein (PNABio, Thousand Oaks, CA, USA) complexed with 80 ng/µL sgRNAs synthesized using the ENGen sgRNA kit (NEB, Ipswitch, MA, USA). Embryos were collected from hypergravid females over a 15 min period and then manually aligned using a fine spotting paint brush, transferred to double-face Scotch tape (Scotch Brand, St. Paul, MN, USA), and covered with Halocarbon 27 oil (Millipore Sigma, St. Louis, MO, USA). No later than 30 min after collection, preblastoderm embryos were then injected using a Femtojet microinjector (Eppendorf, Hamburg, Germany) set to a constant injection pressure of 600 hPa and a backpressure of 250 hPa. The Halocarbon 27 oil was then immediately washed from the embryos with deionized water, and the embryos were allowed to develop for 16–24 h in a humid Petri dish prior to genomic DNA extraction using DNAzol (Thermo Fisher Scientific, Waltham, MA, USA). PCR products spanning the sgRNA target sites were then amplified using primers BR-20 and BR-23 (Table S1), gel purified using the Zymo gel purification kit (Zymo Research, Irvine, CA, USA), Sanger sequenced at the University of Missouri DNA Core (Columbia, MO, USA), and assessed for trace sequence decay using the Inference of CRISPR Edits (ICE) tool from Synthego (Synthego Performance Analysis, ICE Analysis. 2019. v2.0. Synthego).

### 2.3. Construction of Donor Plasmid DNAs

To initially test whether the Chr2:32138225 locus can be successfully targeted via homology-directed DNA repair using CRISPR/Cas9, we designed a donor plasmid containing the enhanced cyan fluorescent protein (ECFP) coding sequence under control of the photoreceptor-specific 3xP3 promoter [33,34]. The transcription terminator originated from the large T-antigen encoding gene of SV40. This expression cassette was then flanked by an upstream homology arm amplified from the HWE strain of *Ae. aegypti* containing the genomic sequence upstream of the CRISPR/Cas9 target site (1238 bp; Chr2:321380975-321382213) and a downstream homology arm containing the genomic sequence downstream of the CRISPR/Cas9 target site (1743 bp; Chr2:321382225-321383968).

The anti-ZIKV effector DNA construct was based on this eye marker construct into which the IR effector expression cassette was inserted. The IR molecule consisted of 538 bp cDNA sequences derived from the ZIKV NS3/4A encoding region in sense and antisense orientations [29], which were separated by the small *sialokinin1* intron [35]. The IR molecule was placed under control of the bloodmeal inducible, midgut-specific *Ae. aegypti* carboxypeptidase A promoter (*AeCpA*) [19,36]. The same transcription terminator and homology arms as described above were used for this construct, now containing eye marker and IR effector.

Plasmid construction was performed based on a combination of conventional restriction enzyme-mediated cloning and Gibson assembly-based cloning. The annotated sequences for both DNA constructs, the eye marker-based reporter and the complete anti-ZIKV effector construct, are available at NCBI under accessions MT926371 and MT926370, respectively. All primer sequences and the gBlock sequence for the anti-ZIKV effector and small *sialokinin1* intron are provided in supplemental Table S1.

### 2.4. Establishment of a Transgenic Line of Ae. aegypti Containing an Anti-ZIKV IR Effector

Donor plasmids were isolated using the Zymo plasmid midiprep kit (Zymo Research, Irvine, CA, USA) and added at a final concentration of 80 fmol/µL to an injection mix containing 300 ng/µL Cas9-NLS (PNABio), 80 ng/µL sgRNA, and 100 ng/µL ku70 dsRNA. Preblastoderm embryos were collected from hypergravid *Ae. aegypti* females (HWE strain) over a 15-min time period, aligned using a fine spotter paint brush for an additional 20–30 min, then transferred to double-face Scotch brand tape and covered with Halocarbon 27 oil (Sigma-Aldrich, St. Louis, MI, USA). The posterior ends of the embryos were then injected following the same methodology as used for the sgRNA activity testing. The oil was rinsed off and the embryos were transferred to moistened Kimwipe tissue and allowed to develop for 7 days prior to hatching. Surviving G0 males were individually outcrossed to 7–10 virgin HWE females, allowed to mate for 5 days, then pooled. Surviving G0 females were mass-crossed to an equal number of HWE males and also allowed to mate for 5 days. Outcrossed pools were provided with three subsequent bloodmeals (defibrinated sheep blood, Colorado Serum Co.) and allowed to lay the eggs of the G1 generation. The G1 generation was subsequently hatched and screened for the presence of the ECFP marker and survivors were individually outcrossed a second time to HWE *Ae. aegypti* and then screened by PCR to confirm transgene integration into the Chr2:321382225 locus. Genomic DNA was extracted from whole mosquitoes using DNAzol (Thermo Fisher Scientific, Waltham, MA, USA). Transgene integrity and integration were confirmed by generating three different PCR amplicons—1. spanning the left homology arm (BR-20) and the *sialokinin1* intron (BR-347); 2. spanning the *AeCpA* promoter (BR-348) and the *sialokinin1* intron (BR-345), and 3. spanning the *sialokinin1* intron (BR-223) and ECFP (BR-348)—using Thermo Fisher Superscript II DNA polymerase under the following cycling conditions: 98 °C for 3 min followed by 35 cycles of 98 °C for 20 s, 62 °C for 20 s, 72 °C for 1 min, and a final extension step of 72 °C for 5 min. Critically, the ramping rate for annealing was increased to 3 °C per second, which allowed for PCR product formation to proceed without impairment by the hairpin. DNA sequences of the amplicons were confirmed by Sanger sequencing. All primers sequences are listed in Table S1.

Finally, the transgenic anti-ZIKV effector harboring mosquitoes (ECFP marker) were outcrossed to another transgenic line of *Ae. aegypti* (HWE strain) containing a 3xP3-mCherry (red) eye marker also integrated into the Chr2:321382225 locus. The F1 progeny were then screened to obtain individuals containing both red and blue eye markers; a subset of the heterozygous individuals was used for small RNA profiling, while remaining heterozygotes were reciprocally crossed and screened to obtain true single-locus homozygotes (blue-eye marker only). Homozygosity was confirmed in a sample of the resulting line by outcrossing transgenic males to wild-type virgin HWE *Ae. aegypti*.

### 2.5. Small RNA Sequencing

Adult female mosquitoes were fed a non-infectious artificial bloodmeal approximately 4 days post-emergence. Midguts were dissected 24 h later, cleaned of blood, and placed in TRIzol (Thermo Fisher Scientific, Waltham, MA, USA) for RNA extraction following the manufacturer’s instructions. Each sample was a pool of 15 cleaned midguts/sequencing library. Total RNA samples were then sent to the University of Missouri DNA Core, where TruSeq small RNA libraries were prepared and subjected to Illumina NextSeq Mid Output SE75 deep sequencing. Small RNA sequencing analyses were performed using a pipeline developed in-house by Dr. Greg Ebel’s lab [37]. The small RNA data discussed in this publication have been deposited in NCBI’s Gene Expression Omnibus [38] and are accessible through GEO Series accession number GSE156825 (https://www.ncbi.nlm.nih.gov/geo/query/acc.cgi?acc=GSE156825). The HWE small RNA dataset is presented under accession number GSM4745099 and the anti-ZIKV-NS3/4A small RNA dataset is presented under accession number GSM4745100.

### 2.6. Virus Challenge Experiments

ZIKV isolates used in this study were PRVABC59 of the Asian lineage (accession number KU501215) and Dakar 41525 of the African lineage (accession number KU955591). ZIKV was propagated in Vero cells at a 0.01 multiplicity of infection (m.o.i.) for 72 h using Dulbecco’s modified Eagle medium (DMEM) supplemented with inactivated 3% fetal-bovine serum (FBS). Infected cells were then pelleted, resuspended in cell culture media, and added to defibrinated sheep blood (Colorado Serum Co.) at a 1:1 (vol/vol) ratio. Mosquitoes were fed for ~1 h using an artificial membrane feeder that maintained ~1–2 mL blood-virus mixture at 37 °C for each carton. Engorged females were visually selected after feeding and were maintained in 64 oz. cartons supplied with sucrose and water until further analysis.

### 2.7. Mosquito Tissue Plaque Assays for ZIKV Detection

Tissue samples were homogenized in 500 μL (midguts or salivary glands) or 1000 μL (carcasses) DMEM (7% inactivated FBS, 1% penicillin/streptomycin, 1% glutamine, 1% non-essential amino acids). Each sample was then passed through a 0.2 μm Acrodisc Syringe Filter fitted with Supor Membrane (Pall Life Sciences, East Hills, NY, USA). Vero cells were seeded in 24-well plates and were left for three days to achieve confluence. Cells were infected with 10-fold serial dilutions of the homogenates (up to 1/10^5^ PFU/mL) for 1 h at 37 °C. After infection, 1 mL of a sterilized 1% agarose solution containing a nutrient supplement (10% 1× Medium 199 (Sigma-Aldrich), 5% inactivated FBS, 4% sodium bicarbonate, 2% diethylaminoethyl (DEAE)-dextran, 0.5% MEM amino acids (Mediatech Inc., Manassas, VA, USA), 0.5% MEM vitamins) was overlaid on each well. Plates were left to solidify for ~1 h and were then moved to the 37 °C incubator for 6 days. To visualize plaques, 150 μL of 3-(4,5-dimethylthiazol-2-yl)-2,5-diphenyltetrazolium bromide (MTT, 3 mg/mL in 1× phosphate-buffered saline [PBS]) was added to each well followed by ~24 h incubation. Plaques were visually quantified the next day. Viral titers of each sample were calculated as plaque-forming units per milliliter (PFU/mL).

### 2.8. Intrathoracic Inoculation of ZIKV

Mosquitoes were intrathoracically inoculated with ZIKV as described previously [39]. Three-day-old females were anesthetized at 4 °C and inoculated with 100 PFU of virus suspended in a 69 nL volume of growth medium. Two days later, a subset of inoculated mosquitoes was exposed to artificial bloodmeals consisting of defibrinated sheep blood (Colorado Serum Co.) and 10 mM ATP, while the other group of mosquitoes was maintained on a sugar diet. Eight days post-virus injection, whole mosquitoes were processed for plaque assay as described above.

### 2.9. ZIKV Transmission Assays

Saliva was collected from female mosquitoes at 14 dpi as previously described [40,41]. Legs and wings were removed from the mosquitoes, and the proboscises were inserted into a 1 µL capillary (microcaps, Drummond Scientific Company, Broomall, PA, USA) filled with immersion oil type B. Mosquitoes were allowed to salivate into the oil at room temperature for 1 h. The oil containing the saliva was expelled under pressure into 1.5 mL Eppendorf tubes containing 300 µL DMEM medium (20% FBS, 1% penicillin/streptomycin, 1% glutamine, 1% non-essential amino acids) and flash frozen on dry ice. Capillaries were visually analyzed for the presence of saliva, and capillaries that did not contain trace amounts of saliva were discarded. Following salivation, salivary glands were dissected from the same mosquitoes and placed into 1.5 mL Eppendorf tubes containing 500 µL DMEM medium (20% FBS, 1% penicillin/streptomycin, 1% glutamine, 1% non-essential amino acids). Corresponding carcasses were also collected. Saliva, salivary glands, and carcasses were frozen at −80 °C and were processed for plaque assay as described above. Saliva samples were not filtered before cell infection.

### 2.10. Immunofluorescence Assays to Detect ZIKV Antigen

Dissected tissues were fixed in 4% paraformaldehyde and permeabilized with 0.2% Triton X-100. Immunofluorescence assays were performed using the monoclonal antibody 4G2 (1:200 in PBS) targeting a conserved epitope of the flavivirus E protein, as well as using a ZIKV NS1-specific mouse monoclonal antibody (1E11, Immune Technology Corporation, 1:200 in PBS). Anti-mouse IgG, biotinylated species-specific whole antibody from sheep (Amersham BioSciences, Cat. # RPN1001V1), was used as secondary antibody (1:200 in PBS, supplemented with 1% Evan’s blue counterstain). Detection was achieved by addition of Streptavidin-Fluorescein conjugate (Amersham Biosciences, Cat. # RPB1232V1; 1:200 in PBS). Slides were mounted using Mowiol (10%) supplemented with DABCO (1,4-diazobicyclo-[1.2.2]-octane) and visualized with an Olympus BH2 microscope.

### 2.11. Statistical Analyses

All statistical analyses were performed with GraphPad Prism (version 8, LaJolla, CA, USA). Comparisons of virus titers were performed using the non-parametric Mann–Whitney U-test, excluding uninfected mosquitoes. A two-tailed Fisher’s exact test was used to compare infection prevalence. Significance was defined as *p* < 0.05.

## 3. Results

### 3.1. Generation of Transgenic Ae. aegypti Expressing a ZIKV-Specific IR Effector

**Figure 1:**
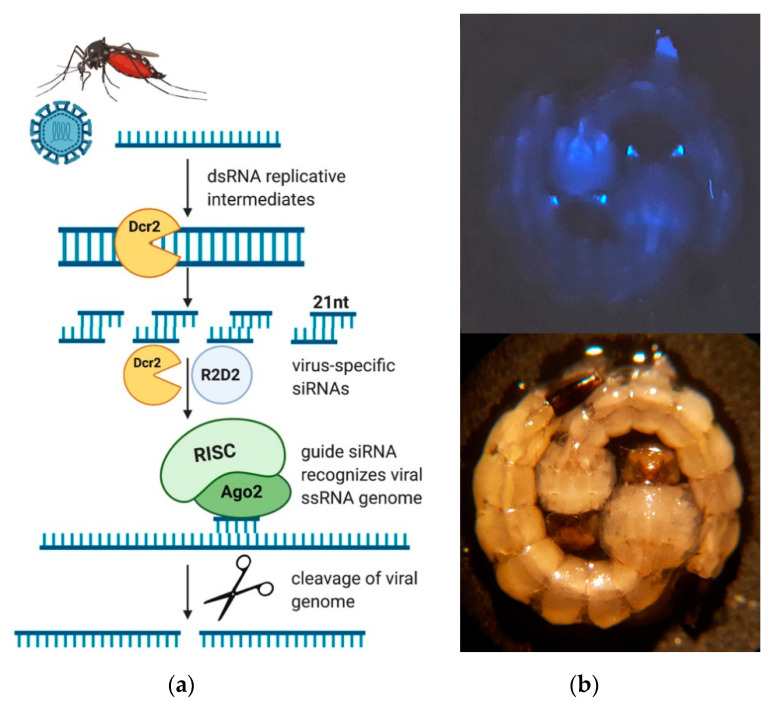
Transgene-mediated resistance to ZIKV in *Ae. aegypti*. (**a**) Schematic outline depicting the siRNA-dependent antiviral pathway in *Ae. aegypti*. Single-stranded RNA (ssRNA) viruses form double-stranded RNA intermediates during replication. Dicer-2 processes dsRNAs into 21nt virus-specific siRNAs. The guide siRNA strand associates with the RISC complex and escorts the endonuclease Ago-2 to complementary sequences in the cell. Ago-2 cleaves the viral ssRNA genome in a sequence-specific manner. Figure created with biorender.com. (**b**) (**top**) Transgenic mosquito larvae expressing ECFP from the photoreceptor-specific promoter 3xP3, enabling marker-based identification by fluorescent microscopy and selection of transgenic individuals. (**bottom**) Transgenic larvae under white light. Dcr2 = dicer-2; RISC = RNA induced silencing complex; Ago2 = Argonaute-2.

**Figure 2:**
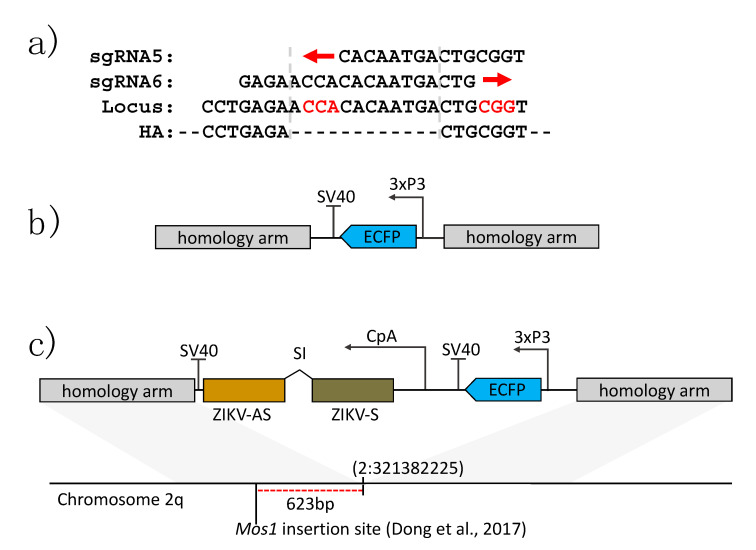
Schematic illustrations of sgRNAs and plasmids used in this study for CRISPR/Cas9 site-specific insertion. (**a**) Nucleotide sequences of sgRNAs (sgRNA5 and sgRNA6), their genomic target sequence, and the junctions of the flanking homology arms (HA) used for transgene integration into the Chr2:321382225 site. The red arrows indicate the directionality of the protospacers, while the respective PAM sequences are displayed in red text. (**b**) 3xP3-ECFP-SV40 donor used to test CRISPR/Cas9 mediated insertion efficiency. (**c**) ZIKV-targeting IR construct. CpA = *carboxypeptidase A* promoter, ECFP = enhanced cyan fluorescent protein, 3xP3 = eye-specific promoter, SV40 = large T antigen terminator from simian virus 40 used as a polyadenylation signal, ZIKV-AS = antisense cDNA of the anti-ZIKV IR effector, ZIKV-S = sense cDNA of the anti-ZIKV IR effector, SI = small intron of the *Ae. aegypti sialokinin1* gene.

Based on our previous success in generating DENV2 resistance [19,20,21], we aimed to engineer ZIKV-resistant *Ae. aegypti* by expressing a ZIKV-specific IR RNA sequence in vivo, intended to trigger the mosquito’s antiviral RNAi pathway (Figure 1a). The IR sequence (Figure S1) was chosen based on previously published data that had identified the NS3/4A region of the viral genome to be highly conserved amongst different ZIKV strains and a robust RNAi target [29]. In order to identify the optimal site for Cas9-mediated transgene insertion around locus Chr2:321382225, we first injected three replicates of ~100 *Ae. aegypti* embryos with injection mixes containing 300 ng/µL Cas9 protein complexed with 80 ng/µL of sgRNA. Injected embryos were allowed to develop for 16–24 h, after which we extracted their genomic DNA and amplified it across the target locus to assess for indels using Sanger sequencing and the Synthego ICE tool. Overall, two sgRNAs (#5 and #6, Table S2) demonstrated activity (Table 1), while the two other sgRNAs demonstrated no activity. The two sgRNAs that were active overlapped each other on the sense and antisense strands of the genomic DNA, respectively, and were located approximately 600 bp downstream of the *mariner Mos1* insertion site in the previously generated transgenic line “Timp-P4” [28]. A common donor was constructed for both sgRNAs that contained a 7 bp gap from the predicted cut site of sgRNA 5 and a 12 bp gap from the cut site of sgRNA 6 (Figure 2a,b). This plasmid sequence has been deposited into GenBank under the accession number MT926371.

Following the construction of the donor plasmid (Figure 2c), we injected *Ae. aegypti* embryos with the same Cas9/sgRNA concentration used for the sgRNA efficacy assessment along with 80 fmol/µL donor plasmid and 100 ng/µL of ku70 dsRNA [42] to suppress non-homologous end-joining events. Surviving G0 males and females were individually outcrossed to HWE, provided three bloodmeals, and assessed for transformation rates (Table 1). One pool was positive for site-specific insertion when sgRNA 5 was used, which was then selected for future experiments utilizing the IR effector construct.

Following successful insertion of an ECFP marker construct into the *Ae. aegypti* Chr2:321382225 locus, we used the same methodology to site-specifically insert the anti-ZIKV IR construct. A total of 1510 preblastoderm embryos were injected with injection mixes containing 300 ng/µL Cas9 protein, 80 ng/µL of sgRNA, 80 fmol/µL donor plasmid, and 100 ng/µL of ku70 dsRNA (to silence non-homologous end-joining events [42]), yielding 184 G0 survivors (12%), which were outcrossed to HWE to obtain the G1 population. In the G1 generation, 37 individuals displayed eye-specific ECFP expression within one of the male pools (consisting of ~200 virgin *Ae. aegypti* that had been allowed to individually mate with ~20 of the surviving G0 males). Transgenic individuals, hereafter termed “anti-ZIKV-NS3/4A,” were again outcrossed to HWE to obtain the G2 generation. The transgene insertion locus and effector sequence were confirmed via Sanger sequencing, and the population was genetically balanced with the help of a 3xP3-mCherry marker inserted into the Chr2:321382225 site (unpublished) to obtain a homozygous population (Methods, Section 2.4). The anti-ZIKV IR construct sequence has been deposited in GenBank under the accession number MT926370.

### 3.2. The ZIKV-Specific IR Effector Is Processed by the Endogenous siRNA Machinery of the Mosquito

**Figure 3:**
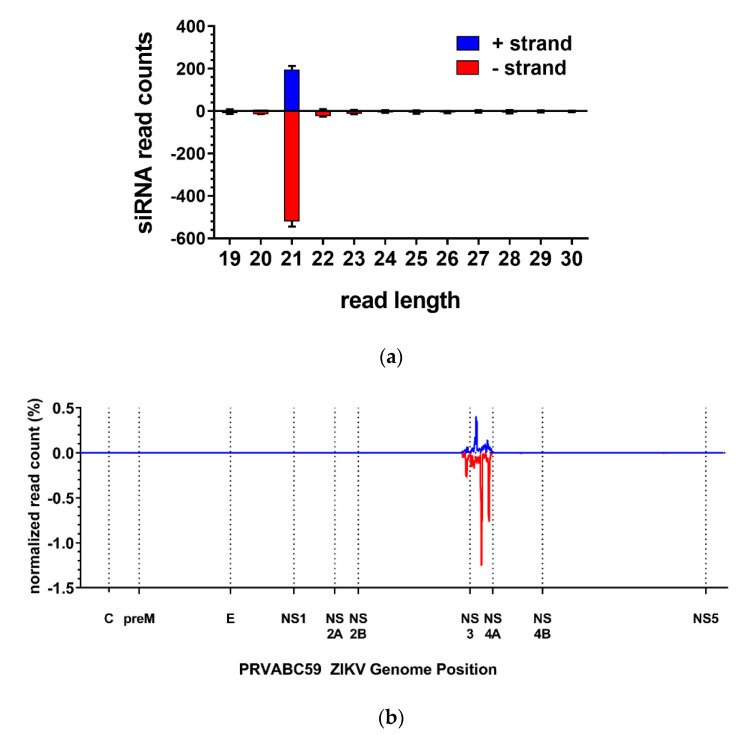
The anti-ZIKV-NS3/4A IR effector is processed by the midgut’s RNAi machinery. (**a**) Anti-ZIKV-NS3/4A mosquitoes process the IR effector into 21 nt ZIKV-specific siRNAs. Black bars indicate mean with range error bars. (**b**) Positional analysis of ZIKV-specific siRNAs reveals that they are complementary to the NS3/4A region of the ZIKV (PRVABC59) genome, which is the region that is targeted by the transgene.

To confirm that the anti-ZIKV-NS3/4A IR was successfully processed by the mosquito’s siRNA machinery, we performed small RNA sequencing on midguts at 24 h post-non-infectious bloodmeal (pbm). ZIKV-specific 21 nucleotide (nt) siRNAs were abundantly detected in midguts of the transgenic mosquitoes at 24 h pbm, but not in the midguts of the HWE control (Figure 3a). The exact size of 21 nt indicates that the NS3/4A IR was processed by the endogenous RNAi pathway. Further positional analysis revealed that the ZIKV-specific siRNAs aligned exclusively to the NS3/4A region, which was specific for the IR effector (Figure 3b). These data indicate that the ZIKV IR was successfully recognized and processed by the RNAi machinery in the midgut of females at 24 h pbm.

### 3.3. Ae. aegypti Expressing the Anti-ZIKV IR Effector Are Resistant to ZIKV

**Figure 4:**
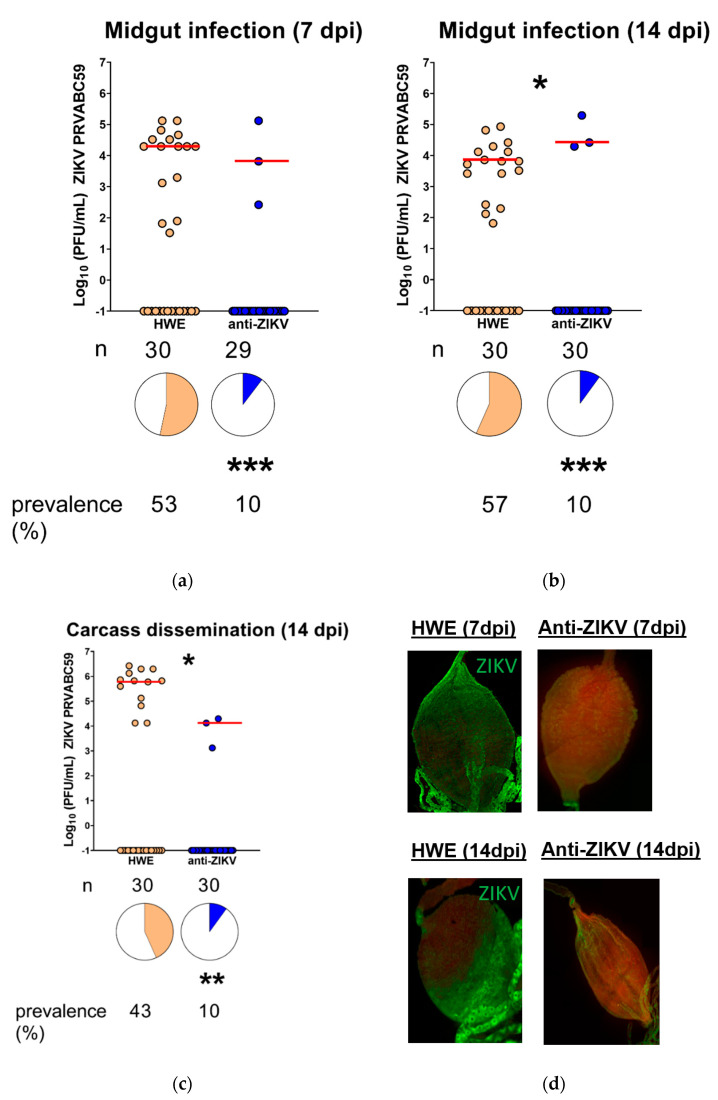
ZIKV IR effector expressing mosquitoes (anti-ZIKV-NS3/4A) are resistant to ZIKV. Infected midguts at (**a**) 7 dpi or (**b**) 14 dpi. (**c**) Infected carcasses showing disseminated infection at 14 dpi. (**d**) Representative immunofluorescence assay images of midguts obtained from HWE and anti-ZIKV-NS3/4A mosquitoes at 7 dpi (top) or 14 dpi (bottom). Primary antibodies recognized the ZIKV E and NS1 proteins. Red bars indicate median virus titers. Stars above graph compare virus titers between infected groups. Stars below pie charts compare infection prevalence; n = number of mosquitoes tested; * = *p* < 0.05, ** = *p* < 0.01, *** = *p* < 0.001. “Anti-ZIKV” = anti-ZIKV-NS3/4A (transgenic) mosquitoes.

To test if the anti-ZIKV-NS3/4A mosquitoes were resistant to ZIKV, we challenged them, along with the parental non-transgenic HWE control, with 3 × 10^5^ plaque-forming units (PFU)/mL ZIKV PRVABC59. We dissected midguts at 7 and 14 dpi and performed plaque assays on these tissues to determine virus titers present in the transgenic versus the HWE control mosquitoes. At 7 dpi, three (10%) transgenic midguts were infected with the virus as compared to 53% of HWE control midguts (*p* = 0.0006, Fisher’s exact test) (Figure 4a). The three transgenic mosquito midguts that were ZIKV infected displayed similar median titers as the HWE midguts that were infected (*p* = 0.9329, Mann–Whitney U-test). At 14 dpi, again, three (10%) of the transgenic midguts were infected as compared to 57% of the HWE control midguts (*p* = 0.0003, Fisher’s exact test) (Figure 4b), exhibiting similar, albeit slightly higher, median titers (*p* = 0.0377, Mann–Whitney U-test).

To measure virus dissemination, we also performed plaque assays on matching carcasses at 14 dpi. We found that the transgenic carcasses that matched the infected transgenic midguts were also infected but showed significantly lower median titers when compared to the HWE carcasses (*p* = 0.0232, Mann–Whitney U-test) (Figure 4c). Overall, 10% of the anti-ZIKV-NS3/4A carcasses were infected as compared to 43% of the HWE control carcasses (*p* = 0.0074, Fisher’s exact test) (Figure 4c). We also performed immunofluorescence assays on midguts dissected at both time points from both the HWE and anti-ZIKV-NS3/4A groups using monoclonal antibodies that bind to the E and NS1 ZIKV proteins. At both time points, all HWE midguts that were imaged showed the presence of viral antigen, while none of the transgenic midguts imaged did so (Figure 4d).

**Figure 5:**
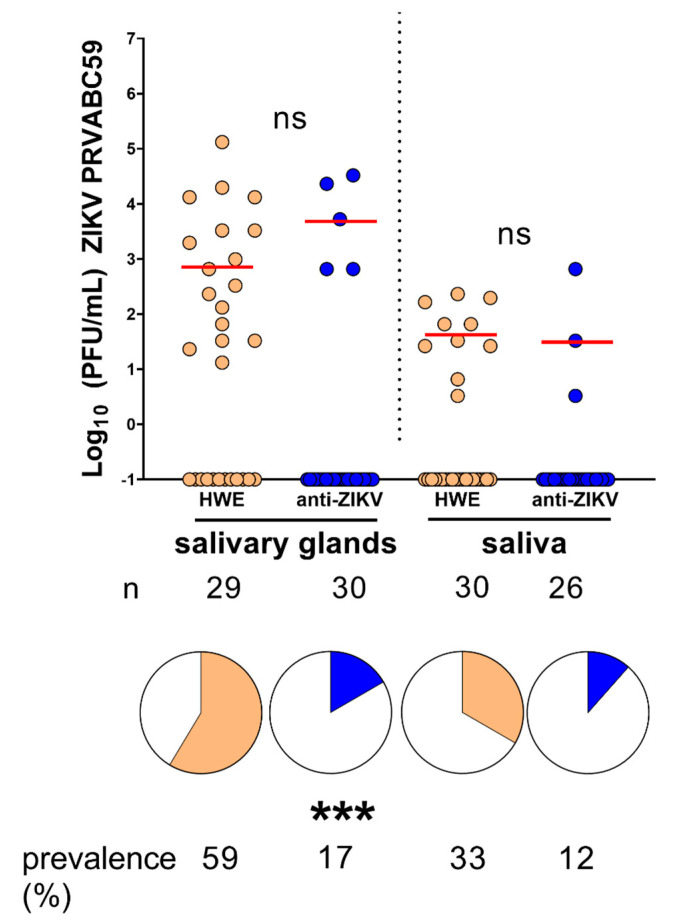
Transgenic anti-ZIKV-NS3/4A mosquitoes block ZIKV transmission. Infected salivary glands (**left**) or saliva (**right**) at 14 dpi. Red bars indicate median virus titers. “ns” above graph compares virus titers between infected groups. Stars below pie charts compare infection prevalence; n = number of mosquitoes tested; n = number of mosquitoes tested; ns = not significant; *** = *p* < 0.001.

To determine whether the anti-ZIKV-NS3/4A mosquitoes could block virus transmission, we performed transmission assays by titrating mosquito salivary glands and saliva at 14 dpi. We challenged HWE and anti-ZIKV-NS3/4A mosquitoes with bloodmeals containing 7 × 10^5^ PFU/mL of ZIKV PRVABC59. We found that five transgenic mosquitoes (17%) harbored infectious virus in their salivary glands, three of which also displayed saliva containing virus. In comparison, 59% of controls had infected salivary glands (*p* = 0.001, Fisher’s exact test) and 33% released saliva containing virus (Figure 5). When only considering infected mosquitoes of both the transgenic and control groups, there were no significant differences between viral titers in the salivary glands (*p* = 0.1338, Mann–Whitney U-test) or in the saliva (*p* > 0.9999, Mann–Whitney U-test). These results show that the anti-ZIKV-NS3/4A mosquitoes are able to significantly block virus replication in their salivary glands, which leads to a decrease in virus prevalence in saliva.

### 3.4. Ae. aegypti Expressing the Anti-ZIKV IR Effector Lose Their Resistance to the Virus When Their Midgut Infection Barriers Are Bypassed

**Figure 6:**
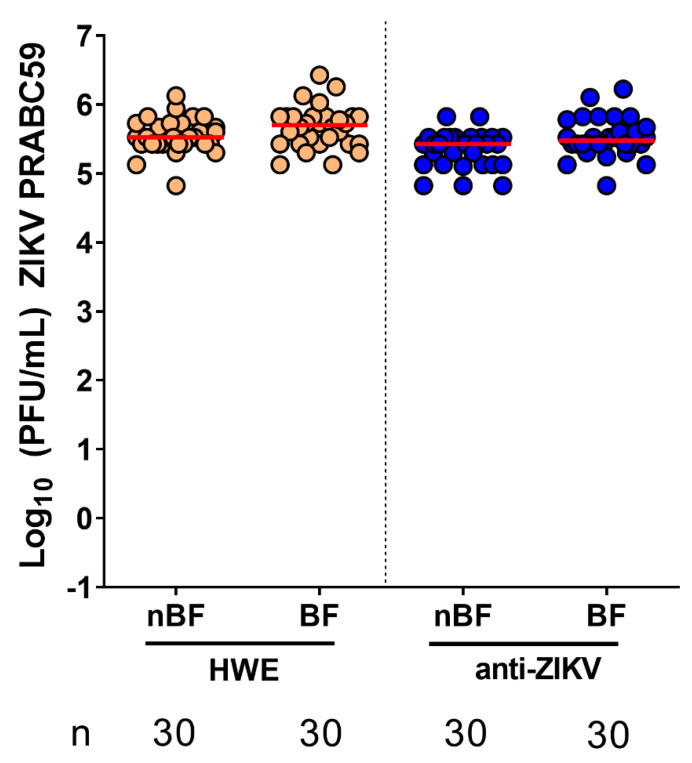
Anti-ZIKV-NS3/4A transgenics lose virus resistance when their midgut infection barrier is bypassed. After intrathoracic virus injection, both HWE mosquitoes (**left**) and anti-ZIKV-NS3/4A (“anti-ZIKV”) transgenic mosquitoes (**right**) either received a non-infectious bloodmeal (to induce expression of the transgene) or a sugarmeal. Red bars indicate medians. nBF = non-bloodfed (sugarmeal), BF = bloodfed, n = number of mosquitoes tested.

We next sought to confirm whether the ZIKV resistance observed in the anti-ZIKV-NS3/4A transgenic mosquitoes was due to transgenic expression of the IR effector in the midgut, the initial site of infection after a mosquito ingests a viremic bloodmeal. We therefore hypothesized that by bypassing the IR-mediated midgut infection barrier, the transgenic mosquitoes would lose virus resistance. To bypass this barrier, we intrathoracically injected both HWE and anti-ZIKV-NS3/4A mosquitoes with 100 PFU ZIKV (PRVABC59). By infecting mosquitoes through artificial means—intrathoracic inoculation—as opposed to a more natural route of infection—bloodfeeding—we could assess the efficacy of the transgene-induced midgut infection barrier. After intrathoracic inoculation, we then separated both mosquito types into two groups: one that would receive a sugarmeal and another group that would receive a non-infectious bloodmeal (to trigger expression of the anti-ZIKV-NS3/4A IR). We then performed plaque assays on whole mosquitoes at 8 days post-virus injection. Regardless of bloodfeeding status, all mosquitoes became infected with ZIKV at high titers (Figure 6). This result indicates that the ZIKV resistance phenotype displayed by the anti-ZIKV-NS3/4A mosquitoes is caused by the transgenically imposed midgut infection barrier.

### 3.5. Resistance of Anti-ZIKV-NS3/4A Transgenics to ZIKV Shows a Tendency to Be Virus Strain-Specific

**Figure 7:**
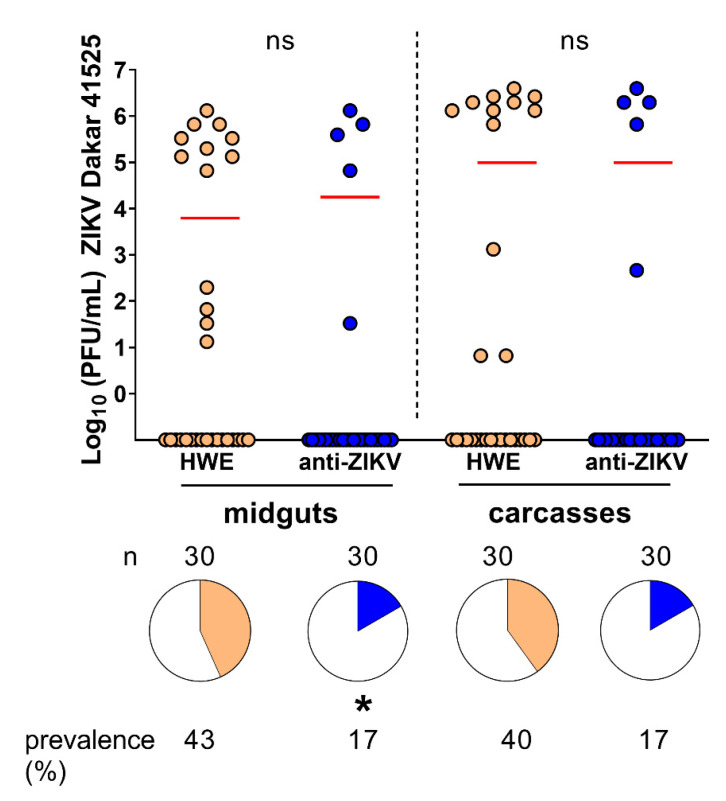
Anti-ZIKV-NS3/4A mosquitoes are significantly resistant to ZIKV Dakar 41525 in their midguts. Infected midguts (**left**) or carcasses (**right**) of anti-ZIKV-NS3/4A (“anti-ZIKV”) and HWE mosquitoes at 14 dpi after infection with ZIKV Dakar. Red bars indicate median virus titers. “ns” above graph refers to virus titers between infected groups. Stars below pie charts compare infection prevalence; n = number of mosquitoes tested, ns = not significant; * = *p* < 0.05.

The endogenous endonuclease Argonaute-2 slices RNAs that are complementary to the guide siRNA in a sequence-specific manner. We therefore asked whether the anti-ZIKV-NS3/4A transgenics would be resistant to any ZIKV strains that differed in their nucleotide sequences encoding the NS3/4A region of the viral genome. We aligned the anti-ZIKV-NS3/4A IR (derived from the PRVABC59 strain of the Asian lineage) to the homologous region of a different ZIKV strain (Dakar 41525 of the African lineage) and found that the Dakar 41525 strain was 89% identical to the transgenic IR effector. We next challenged HWE and anti-ZIKV-NS3/4A mosquitoes with 6 × 10^4^ PFU/mL ZIKV Dakar 41525 and collected midguts and carcasses at 14 dpi for plaque assays. We found that significantly fewer anti-ZIKV-NS3/4A midguts were infected (*p* = 0.047, Fisher’s exact test) as compared to the HWE controls, and all infected transgenic midguts led to virus dissemination in the corresponding carcasses (Figure 7). Similar to ZIKV PRVABC59, the ZIKV Dakar 41525-infected anti-ZIKV-NS3/4A mosquitoes showed viral titers that were similar to the viral titers of the HWE control, in both the midguts (*p* = 0.5191, Mann–Whitney U-test) and the carcasses (*p* = 0.7778). Although we observed evidence of resistance against ZIKV Dakar 41525, the anti-ZIKV-NS3/4A mosquitoes appeared to be slightly more susceptible to a ZIKV strain that was not identical in sequence to the effector cargo. These results imply that the transgenic IR effector may be less protective against ZIKV strains that differ in their viral genome sequence by more than 10% when compared to the virus-derived sequence of the transgene.

## 4. Discussion

Building from our previous success in generating DENV2-resistant *Ae. aegypti*, in this study, we sought to engineer ZIKV-resistant *Ae. aegypti* that would express a ZIKV-derived long dsRNA. We inserted the IR effector-containing transgene into a specific locus on chromosome 2q using CRISPR/Cas9-mediated site-specific insertion. We showed that the Chr2:321382225 locus can be used to reliably insert transgenes via CRISPR/Cas9 technology with as few as ~700 embryos needed to be injected in order to obtain a transgenic line. Site-specific transgene integration allows for the efficacy of various anti-ZIKV effectors to be compared side-by-side without being confronted with varying position effects. In addition, the ability to balance the genomic insertion locus with dominant markers expressing different fluorescent proteins allowed us to obtain homozygous populations within two genetic crosses.

Once we established the transgenic anti-ZIKV-NS3/4A line of mosquitoes, we challenged them with two ZIKV strains to test the efficacy of the IR effector to silence the viruses. We found that 90% of the transgenic mosquitoes blocked ZIKV (PRVABC59) infection in their midguts at 7 and 14 dpi, which also prevented disseminated infections. More than 80% of these mosquitoes released no virus in their saliva and showed no infections in their salivary glands as measured by transmission assays. We confirmed that the observed resistance was due to the midgut infection barrier induced by the transgene, which was expected since the *CpA* promoter is tissue-specific for the midgut [27]. Our RNAi-based strategy, however, may have been sequence-specific, or at least dependent on ZIKV replication dynamics; significantly fewer transgenic mosquito midguts (the tissue in which the transgene is expressed) became infected with a heterologous ZIKV strain as compared to controls, while marginally fewer transgenic mosquito carcasses were infected as compared to controls.

The anti-ZIKV-NS3/4A sequence expressed in our transgenic mosquito line was selected based on previous findings in which mosquitoes were highly protected against ZIKV (PRVABC59) when intrathoracically injected (prior to virus infection) with dsRNAs derived from the viral sequence [29]. The anti-NS3/4A sequence (in Magalhaes et al. (2019), this same sequence is termed “dsZIKV5”) overlaps with the last 183 bp of the NS3 region and 355 bp of the NS4A region (nearly the entire NS4A sequence) of ZIKV PRVABC59. In Magalhaes et al. (2019), mosquitoes were intrathoracically inoculated with 250 ng of five different long dsRNAs targeting ZIKV, including the anti-ZIKV-NS3/4A dsRNA [29]. Three days later, all mosquito groups, including three control groups, received an artificial virus-containing bloodmeal to assess virus resistance [29]. Only one mosquito injected with the anti-ZIKV-NS3/4A dsRNA was infected at 7 dpi, and no mosquitoes were infected at 14 dpi [29]. These results contrasted with those from our transgenic mosquitoes, where 10% of the mosquitoes consistently became ZIKV infected at 7 or 14 dpi. These differences may have been because the mosquitoes in [29] received a higher dose of the anti-ZIKV/NS3/4A dsRNA by intrathoracic injection as compared to the number of anti-ZIKV-NS3/4A dsRNA molecules expressed in our transgenic mosquitoes. Timing may also be a factor influencing virus resistance—the mosquitoes that received the anti-ZIKV-NS3/4A dsRNA by injection were challenged with ZIKV (PRVABC59) three days later, whereas the anti-ZIKV-NS3/4A transgene is expressed in the mosquito midgut at the time of infection. As a point of similarity, mosquitoes that were injected with the anti-ZIKV-NS3/4A dsRNA were less resistant to divergent ZIKV strains, as we observed here for the transgenic anti-ZIKV-NS3/4A mosquitoes.

It is also worth noting that although Magalhaes et al. (2019) challenged mosquitoes with a higher ZIKV titer (8.7 × 10^6^ PFU/mL) than we did (~10^5^ PFU/mL), the authors used frozen ZIKV stocks, which have been shown to exhibit decreased infectivity when compared to fresh virus cultures [43]. In this study, we consistently challenged both the HWE control and the anti-ZIKV-NS3/4A mosquitoes with freshly cultured ZIKV at ~10^5^ PFU/mL across the different experiments. We aimed to feed similar ZIKV concentrations across replicate experiments because it has been shown that mosquito infection prevalence is highly dependent on infectious virus titers, as well as virus and mosquito strains [44]. It is therefore important that studies like ours and others report the infectious bloodmeal titers, as well as the genetic background of the mosquitoes tested, as these variables may confound the results.

This is the first transgenic line of *Ae. aegypti* engineered to trigger the endogenous siRNA pathway targeting ZIKV. Previously, Buchman et al. (2019) generated ZIKV-resistant transgenic *Ae. aegypti* that expressed synthetic small RNAs, which induced the miRNA pathway [45]. Five of the eight synthetic miRNAs were processed in homozygous mosquitoes, which led to a 100% reduction in ZIKV genome equivalents in midguts, carcasses, and saliva [45]. These miRNAs targeted the capsid, pre-membrane, NS1, and NS5 regions of the ZIKV genome [45]. Given the fact that our results consistently showed that 10% of the transgenic mosquitoes were infected after siRNA targeting of the virus, it might be possible that ZIKV is less susceptible to the siRNA pathway. Indeed, it has been demonstrated that ZIKV subgenomic flaviviral RNA (sfRNA) interacts with specific mosquito proteins and suppresses RNAi in *Ae. aegypti* [46]. This viral immune evasion strategy may be a limiting factor when attempting to enhance RNAi-based immune responses in vivo in an effort to engineer ZIKV-resistance in mosquitoes. Additionally, targeting multiple regions of the ZIKV genome may be more efficacious in viral blocking than just targeting a single long region. The observed discrepancies between the viral silencing efficiencies of siRNAs and synthetic miRNAs could be also caused by different expression patterns of dicer-1 and dicer-2. The siRNA pathway uses dicer-2 to process long dsRNAs into siRNAs; it is possible that dicer-2 is less strongly expressed than dicer-1 and therefore produces proportionally fewer siRNAs as compared to dicer-1-mediated miRNAs.

Although in their respective transgenic *Ae. aegypti* lines, the anti-ZIKV IR effector of this work was processed into siRNAs at a similar rate as an anti-DENV2 IR effector in a previous study [20], we observed lower rates of resistance against ZIKV as compared to DENV2. We consistently observed that 93–100% of transgenic anti-DENV2 IR *Ae. aegypti* are resistant to DENV2 (but not to other serotypes) at 14 dpi regardless of the DENV2 genotype used in the challenge experiment [20]. However, in this study, we found that 83–90% of the anti-ZIKV-NS3/4A mosquitoes are resistant to ZIKV (dependent on the virus strain) at 14 dpi. Furthermore, the anti-ZIKV-NS3/4A mosquitoes that were infected with ZIKV exhibited similar titers as the controls. Because the anti-ZIKV-NS3/4A mosquitoes were homozygous for the transgene, and because we screened all transgenics for fluorescent eye coloration before experiments, we do not believe that the transgenic mosquitoes that remained susceptible to the virus lacked the transgene. Furthermore, given that we have engineered 93–100% DENV2 resistance by this same strategy in *Ae. aegypti* of the same genetic background (HWE), we doubt that this susceptibly is due to genetic differences between mosquitoes in siRNA processing. However, it is possible that even with robust effector transgene expression, the siRNA pathway may become saturated. Future work, driving the expression of arbovirus-specific long dsRNAs under different promoters that allow for transgene production in carcass or salivary gland tissues may also enhance the efficacy of this strategy. Finally, ZIKV may be less susceptible to siRNA targeting as compared to DENV2, which could suggest that viruses of the same family (in this case, *Flaviviridae*) are not equally susceptible to mosquito innate immune mechanisms or various types of antiviral effectors.

Future work aimed at the development of antiviral effectors that target multiple arboviruses synchronously is needed since several arboviruses may co-circulate in a given region. This study was proof-of-concept, but further optimization may improve rates of resistance. For example, engineering mosquitoes to express several long dsRNAs targeting multiple regions of the ZIKV genome, multiple ZIKV strains, or even multiple arboviruses could broaden the transgenic approach explored in this paper. Using complementary antiviral effector strategies in tandem may also provide an avenue toward achieving this goal. For example, in addition to generating ZIKV or DENV resistance by triggering *Ae. aegypti* innate immunity, DENV1-4 resistance has been achieved through transgenic expression of a single chain antibody [47] and CHIKV resistance has been achieved through transgenic expression of an antiviral ribozyme [48]. Furthermore, using multiple strategies will minimize the risk of viruses developing resistance against these effectors. Ultimately, novel vector control techniques targeting *Ae. aegypti* populations will rely on potent antiviral effectors that block virus replication in vivo and, in this way, virus transmission.

## Figures and Tables

**Table 1:** Testing of sgRNAs for genome editing rates in the Chr2:321382225 locus of *Ae. aegypti*.

Injection Mix	Embryos Injected	Male	Female	Survival (%)
sgRNA 5	735	19	22	5.6
sgRNA 6	928	20	23	4.6
sgRNAs 5 + 6	882	38	36	8.4
Anti-ZIKV-IR	1510	84	100	12.1

## Supplementary Materials

The following are available online at https://www.mdpi.com/1999-4915/12/11/1231/s1, Table S1: Primer sequences used in this study, Table S2: Genomic location of the desired target insertion site for the inverted repeat transgene and the locations of the closest sgRNAs that did not contain predicted off-target sites in the *Ae. Aegypti* genome, Figure S1: ZIKV-specific NS3/4A IR sequence.
