# Supplementary material for: The Antiviral Small-Interfering RNA Pathway Induces Zika Virus Resistance in Transgenic Aedes aegypti
Source: Viruses. 2020 Oct 30;12(11):1231. doi: 10.3390/v12111231 (PMC7692394; doi:10.3390/v12111231)
Supplement: Supplementary file 1 [file viruses-12-01231-s001.pdf]

Supplementary Materials: The following are available online at www.mdpi.com/xxx/s1

Table S1. Primer sequences used in this study

**Primer**	**Sequence (5' to 3')**	**Purpose**
BR-4	GAAATTAATACGACTCACTATAGGTG GTTCTCAGGCGACAGTTGTTTTAGAG CTAGAAATAGC	Forward primer for sgRNA 3
BR-5	GAAATTAATACGACTCACTATAGGTA ATGGCACAGGCTATAGGTTTTAGAGC TAGAAATAGC	Forward primer for sgRNA 4
BR-6	GAAATTAATACGACTCACTATAGGAC CAACCGCAGTCATTGTGGTTTTAGAG CTAGAAATAGC	Forward primer for sgRNA 5
BR-7	GAAATTAATACGACTCACTATAGGAG AACCACACAATGACTGGTTTTAGAGCT AGAAATAGC	Forward primer for sgRNA 6
BR-20	AACGAGATGCCTTCTCCTGA	Genomic primer for indel assessment
BR-23	AAAATGGCGTTCGATGAGAC	Genomic primer for indel assessment
BR-51	CATAAGCTTGCTAATCGGTGTCAATTC CATC	Upstream homology arm forward
BR-52	CATGGATCCTAACTCGAGTCTCAGGC GACAGTATGGCTC	Upstream homology arm reverse to introduce a XhoI site
BR-53	CATGGATCCATACTCGAGCTGCGGTT GGTCGTGTATTTGAAG	Downstream homology arm forward to introduce a XhoI site
BR-54	CATCTCGAGAAATTCGAGCTCGCCCG G	3xP3FOR-XhoI forward
BR-55	CATCTCGAGCCGTACGCGTATTCGATA AG	SV40REV-XhoI reverse
BR-73	GGGAAGGTTTCTGGTACAGG	Downstream homology arm reverse
BR-94	CATTATCTCGAGCTTCACGTTTTCCCA GGTCAGAAGC	attP-XhoI forward
BR-10 2	CTGCTATGTGGCGCGGTATTATC	β-lactamase breaking primer for Gibson assembly
BR-103	GATAATACCGCGCCACATAGCAG	β-lactamase breaking primer for Gibson assembly

BR-124	AATCTCGAGCTAGCACTAGTCGCGCTCGCGCG	attP-NheI reverse
BR-125	CAATATCTAGAGCCGTACGCGTATTCGATAAG	SV40-XbaI reverse
BR-126	ACAATTCTAGAGAAATTCGAGCTCGCCCGG	3xP3-XbaI forward
BR-138	TCGAGAATTCGCTAGCACTAGTCGCGCTCGCGCG	attP-NheI-EcoRI reverse
BR-139	CATTATCCGCGGCTTCACGTTTTCCCAGGTCAGAAGC	attP-SacII forward
BR-211	AGAAACCGCGGAGATCTCGGCCGCGACTCTAGATC	SV40-SacII-BglII forward
BR-212	CAATAGCTAGCTAAGATACATTGATGAGTTTGGACAAAC	SV40-NheI reverse
BR-213	AAACCTCTCGAGCGACCAAC	Anti-ZIKV dsRNA trigger + intron-XhoI
BR-214	GGTTTCTGCAGCTGCCTAATGATATATTTTTAAT	Anti-ZIKV dsRNA trigger + intron-PstI
BR-219	ATCGAGGCCTGAGCTCTTACGCGTGCTAGTTTTTG	*Carboxypeptidase A* promoter-StuI forward
BR-220	ATAGTAATCTCGAGCGGCCGCCTGCAG	*Carboxypeptidase A* promoter-XhoI reverse
BR-223	TCGGCATGGACGAGCTGTAC	ECFP anchored primer running out of the end of the color cds
BR-243	CGGCCGCGACTCTAGATC	Intermediate for final Gibson assembly
BR-245	TAGTGATTAAAAATATATCATTAGGCAGCTGATCTTTGCTTTTCTGGCTCAG	ZIKV dsRNA trigger sense strand for final Gibson assembly
BR-250	TCTCCGCGGTTTCTCTAGTGGATCCCCCGGGCGACCAACAACACCATAATGG	ZIKV dsRNA trigger sense strand for final Gibson assembly
BR-248	CCCGGGGGATCCACTAG	Intermediate for final Gibson assembly
BR-345	ATCACTAGCCCGGGCTGCA	*Carboxypeptidase A* anchored primer running into the IR

BR-347	GGAGGTGAGCACCCAATCATCAG	*sialokinin1* intron anchored primer running towards the IR promoter
BR-348	AATCACTAACAGAACTTTGAACAAAATC	*sialokinin1* intron anchored primer running towards the inverted-repeat IR terminator
BR-240	AAACCTCTCGAGCGACCAACAACACCATAATGGAAGACAGTGTGCCGGCAGAGGTGTGGACCAGACACGGAGAGAAAAGAGTGCTCAAACCGAGGTGGATGGACGCCAGAGTTTGTTCAGATCATGCGGCCCTGAAGTCATTCAAGGAGTTTGCCGCTGGGAAAAGAGGAGCGGCTTTTGGAGTGATGGAAGCCCTGGGAACACTGCCAGGACACATGACAGAGAGATTCCAGGAAGCCATTGACAACCTCGCTGTGCTCATGCGGGCAGAGACTGGAAGCAGGCCTTACAAAGCCGCGGCGGCCCAATTGCCGGAGACCCTAGAGACCATAATGCTTTTGGGGTTGCTGGGAACAGTCTCGCTGGGAATCTTCTTCGTCTTGATGAGGAACAAGGGCATAGGGAAGATGGGCTTTGGAATGGTGACTCTTGGGGCCAGCGCATGGCTCATGTGGCTCTCGGAAATTGAGCCAGCCAGAATTGCATGTGTCCTCATTGTTGTGTTCCTATTGCTGGTGGTGCTCATACCTGAGCCAGAAAAGCAAAGATCGGAGGTGAGCACCCAATCATCAGATTTTGTTCAAAGTTCTGTTAGTGATTAAAAATATATCATTAGGCAGCTGCAGAAACC	XhoI/PstI tagged *sialokinin1* intron and anti-ZIKV RNAi trigger sequence used to construct IR

Table S2. Genomic location of the desired target insertion site for the inverted repeat transgene and the locations of the closest sgRNAs that did not contain predicted off-target sites in the *Ae. aegypti* genome.

Location	Embryos injected (total N)	*Ae. aegypti* Liverpool strain equivalent cut site[1]	Δ distance (bp)	Synthego score (average indel% +/ SEM)
Dong et al., 2017 [28]	n/a	2:321382845-321382846	0	n/a
sgRNA3	424	2:321382201	644	0
sgRNA4	282	2:321382958	112	0
sgRNA5	282	2:321382222	623	17.0 ± 2.5
sgRNA6	324	2:321382228	629	43.3 ± 3.4

[1]*Ae. aegypti* genome version AGWG AaegL5, vectorbase.org

Figure S1

>ZIKV-specific NS3/4A IR sequence

CGACCAACAACACCATAATGGAAGACAGTGTGCCGGCAGAGGTGTGGACCAGACACGGAGAGAAAAGAGTGCTC
AAACCGAGGTGGATGGACGCCAGAGTTTGTTCAGATCATGCGGCCCTGAAGTCATTCAAGGAGTTTGCCGCTGGG
AAAAGAGGAGCGGCTTTTGGAGTGATGGAAGCCCTGGGAACACTGCCAGGACACATGACAGAGAGATTCCAGGA
AGCCATTGACAACCTCGCTGTGCTCATGCGGGCAGAGACTGGAAGCAGGCCTTACAAAGCCGCGGCGGCCCAATT
GCCGGAGACCCTAGAGACCATAATGCTTTTGGGGTTGCTGGGAACAGTCTCGCTGGGAATCTTCTTCGTCTTGATG
AGGAACAAGGGCATAGGGAAGATGGGCTTTGGAATGGTGACTCTTGGGGCCAGCGCATGGCTCATGTGGCTCTC
GGAAATTGAGCCAGCCAGAATTGCATGTGTCCTCATTGTTGTGTTCCTATTGCTGGTGGTGCTCATACCTGAGCCA
GAAAAGCAAAGATCAGCTGCCTAATGATATATTTTTAATCACTAACAGAACTTTGAACAAAATCTGATGATTGGGT
GCTCACCTCCGATCTTTGCTTTTCTGGCTCAGGTATGAGCACCACCAGCAATAGGAACACAACAATGAGGACACAT
GCAATTCTGGCTGGCTCAATTTCCGAGAGCCACATGAGCCATGCGCTGGCCCCAAGAGTCACCATTCCAAAGCCCA
TCTTCCCTATGCCCTTGTTCCTCATCAAGACGAAGAAGATTCCCAGCGAGACTGTTCCCAGCAACCCCAAAAGCATT
ATGGTCTCTAGGGTCTCCGGCAATTGGGCCGCCGCGGCTTTGTAAGGCCTGCTTCCAGTCTCTGCCCGCATGAGCA
CAGCGAGGTTGTCAATGGCTTCCTGGAATCTCTCTGTCATGTGTCCTGGCAGTGTTCCCAGGGCTTCCATCACTCCA
AAAGCCGCTCCTCTTTTCCCAGCGGCAAACTCCTTGAATGACTTCAGGGCCGCATGATCTGAACAAACTCTGGCGT
CCATCCACCTCGGTTTGAGCACTCTTTTCTCTCCGTGTCTGGTCCACACCTCTGCCGGCACACTGTCTTCCATTATGG
TGTTGTTGGTCGC
